# Thermal response during erbium laser-assisted removal of ceramic crowns and laminate veneers: a scoping review

**DOI:** 10.1007/s10103-026-05100-2

**Published:** 2026-11-14

**Authors:** Gabriela da Silva Chagas, Marcella Batista Rocha, Eduardo Bresciani, Amjad Abu Hasna

**Affiliations:** 1 Department of Restorative Dentistry, Institute of Science and Technology, Universidade Estadual Paulista (Unesp), São José dos Campos, Brazil; 2 Advanced Restorative Dentistry, School of Dentistry and Medical Sciences, Charles Sturt University, Orange, Australia; 3 Department of Restorative Dentistry, Endodontics Division, Institute of Science and Technology, Universidade Estadual Paulista (Unesp), São José dos Campos, Brazil

**Keywords:** Dental debonding, Dental pulp, Dental veneer, Erbium, Lasers, Heating

## Abstract

This review aimed to systematically map and synthesize the available evidence regarding the thermal effects of laser-assisted debonding of ceramic crowns and laminate veneers on the dental pulp and to identify current knowledge gaps. Following the Joanna Briggs Institute methodology and the PRISMA-ScR guidelines, the scoping review was conducted after preliminary screening identified limited and heterogeneous evidence for a systematic review. PubMed, Scopus, and Web of Science were searched through June 2026. Two independent reviewers screened eligible studies and extracted data on laser parameters, irradiation protocols, restorative characteristics, debonding performance, and thermal outcomes. A total of 63 records were identified, and 10 studies met the eligibility criteria and were included in this scoping review. Er, Cr: YSGG was the most investigated laser, followed by Er: YAG. The included studies evaluated the debonding of lithium disilicate and zirconia laminate veneers and full-coverage crowns. Considerable heterogeneity was observed in laser parameters, ceramic materials, restoration thicknesses, and outcome assessment methods. Intrapulpal temperature change and debonding time were the most frequently reported outcomes, with most studies reporting temperature increases below the commonly cited historical thermal threshold for irreversible pulp damage. Current evidence suggests that erbium laser-assisted debonding can be associated with temperature increases within the pulp chamber, with the magnitude of the thermal response varying according to laser parameters, cooling protocols, and restorative characteristics. However, the lack of standardized experimental protocols, standardized control groups, and clinical evidence prevents definitive conclusions regarding pulpal safety or the establishment of evidence-based clinical recommendations.

**Supplementary Information:**

The online version contains supplementary material available at https://doi.org/10.1007/s10103-026-05100-2.

## Introduction

The growing demand for aesthetic dental treatments has increased the use of ceramic crowns and laminate veneers to restore teeth affected by discoloration, trauma, malformations, and other aesthetic concerns [1, 2]. Consequently, dentists are increasingly required to meet these expectations through the placement of ceramic crowns and laminate veneers, or dental bleaching [3, 4]. Despite patients’ satisfaction, indirect restorations may fail clinically because of several factors [5–7], including inadequate case selection, inaccurate diagnosis and treatment planning, poor communication with the dental laboratory, inappropriate material selection, excessive tooth preparation, inadequate isolation and soft tissue management, and improper cementation procedures. Consequently, clinical reintervention is often required to restore function and aesthetics [8, 9].

Several techniques have been proposed for the debonding of failed ceramic crowns and laminate veneers including conventional burs and high-speed rotary instruments, ultrasonic inserts, and high-power lasers [10–20]. However, the potential impact of these procedures on pulp health and the maintenance of pulp vitality remains an important and yet underexplored concern [21]. This potential impact is affected by several factors including debonding time of a failed restoration, the thermal changes stimulated during the debonding, and the restoration type itself. It is worth noting that the dental pulp has a baseline internal temperature, under physiological conditions, ranging from 34 °C to 35 °C, and an intrapulpal temperature increase exceeding 5.5 °C has traditionally been considered the critical threshold for irreversible pulp damage [22]. However, this value should be interpreted as a historical experimental reference rather than as a clinically validated cutoff for predicting pulpal injury. Experimental studies have also demonstrated that temperature increases may affect the viability of cells within dental tissues and negatively affect the viability of surrounding cells [23].

Erbium lasers, particularly erbium-doped yttrium aluminum garnet (Er: YAG) and erbium, chromium: yttrium-scandium-gallium-garnet (Er, Cr: YSGG) lasers, exhibit promising clinical efficacy as it facilitates debonding of different ceramic crown materials, or even as conditioner prior to ceramic cementation, while minimizing damage to the underlying tooth structure [11, 24]. However, adequate air/water cooling during the debonding procedure is essential to prevent excessive intrapulpal temperature increases. Insufficient cooling may result in temperature elevations up to 11.5 °C above physiological levels, potentially exceeding the threshold associated with irreversible pulp damage [20]. Such findings highlight the importance of evaluating thermal changes during laser-assisted restoration removal rather than assuming that laser use inherently provides a thermal advantage. Furthermore, considerable variability in laser systems and irradiation parameters including output power, pulse energy, pulse duration, and operating mode, in addition to restorative characteristics has resulted in heterogeneous study protocols. This variability makes direct comparisons difficult and limits the establishment of standardized clinical recommendations for safe and effective Er: YAG and Er, Cr: YSGG lasers-assisted debonding [11–20].

The success of indirect restoration debonding should not be defined solely by the ability to remove the restoration efficiently. Preservation of pulp vitality is a fundamental component of treatment success, particularly in vital teeth, as pulpal injury may result in irreversible damage and the need for endodontic therapy. Nevertheless, the literature has largely emphasized the mechanical aspects of debonding procedures, including debonding efficiency, treatment time, and laser parameters, while comparatively little attention has been paid to their biological impact on pulpal tissues. Although clinical reports of pulp necrosis associated with laser-assisted debonding are scarce, the absence of documented failures should not be interpreted as evidence of biological safety. Since thermal insult is considered one of the principal mechanisms by which debonding procedures may affect pulpal health, a comprehensive understanding of intrapulpal temperature changes during laser-assisted restoration debonding is essential to ensure that treatment success encompasses both effective debonding and preservation of pulp vitality.

Although Er: YAG and Er, Cr: YSGG lasers have demonstrated efficacy in ceramic crowns and laminate veneers debonding, evidence regarding the thermal effects generated during this procedure and their potential impact on pulp vitality remains limited. Therefore, this review study aimed to map and synthesize the available evidence on the thermal effects of Er: YAG and Er, Cr: YSGG lasers on the dental pulp during the debonding of ceramic crowns and laminate veneers.

## Materials and methods

### Protocol and study design

This study was initially designed as a systematic review to answer the following research question: Does the Er: YAG and Er, Cr: YSGG lasers induce thermal dental pulp injury during the debonding of ceramic crowns and laminate veneers? Accordingly, the review was structured using the PICO strategy, in which Population was teeth restored with ceramic crowns or laminate veneers; Intervention was Er: YAG and Er, Cr: YSGG lasers-assisted debonding/removal; Comparison was not predefined to avoid restricting comparisons with other debonding techniques or protocols, and Outcome was intrapulpal thermal changes and pulp injury.

However, preliminary screening revealed a limited number of studies addressing this specific question and considerable heterogeneity in study designs, laser parameters, ceramic materials, control groups, and outcome measures. In addition, the available evidence primarily consisted of studies reporting temperature changes rather than direct biological assessment of pulpal injury. Therefore, the review was adapted to a scoping review to systematically map the available evidence and characterize the factors associated with thermal responses during erbium laser-assisted ceramic restoration debonding.

For this, the review methodology was adapted to a scoping review to comprehensively map the available evidence and the Joanna Briggs Institute (JBI) methodology for scoping reviews was followed, and the review was reported according to the PRISMA extension for scoping reviews (PRISMA-ScR) guidelines [25]. The review was subsequently structured according to the PCC strategy, where Population was ceramic crowns and laminate veneers requiring debonding, Concept was the thermal effect associated with Er: YAG and Er, Cr: YSGG lasers irradiation including intrapulpal temperature changes and the laser, cooling, and restorative parameters potentially influencing these effects, and Context was ceramic restoration debonding procedures with emphasis on pulp safety. Based on this approach, the final review question was: What thermal effects have been reported during erbium laser-assisted debonding of ceramic crowns and laminate veneers, and which laser, cooling, and restorative parameters are associated with these thermal responses?

### Eligibility criteria

#### Inclusion criteria

- Original clinical and laboratory studies.
- Articles published in English.
- Studies investigating erbium lasers-assisted debonding/ removal of ceramic crowns or laminate veneers.
- Studies reporting at least one relevant thermal outcome, including intrapulpal temperature changes or other measures of intrapulpal temperature increase during the debonding procedure.
- No restrictions regarding publication date.

#### Exclusion criteria

- Case reports, review articles, opinion papers, editorials, letters, or conference abstracts.
- Studies involving ceramic restoration removal without the use of an erbium laser.
- Studies that did not report a thermal outcome related to the debonding procedure.
- Duplicate publications or secondary publications reporting data from previously published studies.

### Information sources and search strategy

The search strategy was developed through an iterative process using Medical Subject Headings (MeSH) and entry terms related to erbium lasers, ceramic restorations, debonding/removal procedures, and thermal or pulpal outcomes. The initial search strategy was developed by piloting Medical Subject Headings (MeSH) and entry terms in the MEDLINE/PubMed database. All terms that resulted in no yields or duplicated studies were removed. Then, the PubMed finalized strategy was adapted for each Scopus and Web of Science database. A literature search was conducted up to June 15th, 2026. The complete search strings for each database are presented in Table 1.

**Table 1:** The information sources and search strategy

PubMed: resulted in 32 articles
(“tooth“[MeSH Terms] OR “ceramics“[MeSH Terms] OR “lithium disilicate“[Title/Abstract] OR “veneer“[Title/Abstract] OR “laminate“[Title/Abstract]) AND (“lasers, solid state“[MeSH Terms] OR “Er: YAG laser“[Title/Abstract] OR “erbium: YAG laser“[Title/Abstract] OR “laser*“[Title/Abstract]) AND (“crown removal“[Title/Abstract] OR (“debond*“[Title/Abstract] OR “veneer removal“[Title/Abstract] OR “restoration removal“[Title/Abstract])) AND (“Dental Pulp“[MeSH Terms] OR “Pulp Vitality“[Title/Abstract] OR “pulp damage“[Title/Abstract] OR “pulp injury“[Title/Abstract] OR “pulpal temperature“[Title/Abstract] OR “overheating“[Title/Abstract] OR “thermal effects“[Title/Abstract] OR “intrapulpal temperature“[Title/Abstract] OR “dental pulp injuries“[Title/Abstract])
Scopus: resulted in 13 articles
TITLE-ABS-KEY(tooth OR ceramics OR “lithium disilicate” OR veneer OR laminate) AND TITLE-ABS-KEY( “lasers, solid state” OR “Er: YAG laser” OR “erbium: YAG laser” OR laser ) AND TITLE-ABS-KEY(“crown removal” OR debond OR “veneer removal” OR “restoration removal”) AND TITLE-ABS-KEY(“Dental Pulp” OR “Pulp Vitality” OR “pulp damage” OR “pulp injury” OR “pulpal temperature” OR overheating OR “thermal effects” OR “intrapulpal temperature” OR “dental pulp injuries”)
Web Of Science: resulted in 18 articles
TS=(tooth OR ceramics OR “lithium disilicate” OR veneer OR laminate) AND TS=( “lasers, solid state” OR “Er: YAG laser” OR “erbium: YAG laser” OR laser ) AND TS=(“crown removal” OR debond OR “veneer removal” OR “restoration removal”) AND TS=(“Dental Pulp” OR “Pulp Vitality” OR “pulp damage” OR “pulp injury” OR “pulpal temperature” OR overheating OR “thermal effects” OR “intrapulpal temperature” OR “dental pulp injuries”)

The literature search was executed independently by two reviewers (G.S.C. and M.B.R.). An expert professor (A.A.H.) guided the development of the search strategies and tailored them to the specific syntax of each repository, applying no date´s restriction. To ensure newly published evidence was captured during the manuscript preparation, weekly automated search alerts were established across all selected databases according to the outlined search strategy.

### Selection of sources of evidence

A total of 55 publications were identified after the removal of duplicate ones. Two reviewers (G.S.C. and M.B.R.) independently screened the titles and abstracts of all identified studies in accordance with the predefined eligibility criteria. When a title and abstract provided insufficient information to make a definitive decision, the full-text was retrieved for closer examination.

Following the initial title and abstract screening, the full-text articles of the remaining potentially relevant studies were retrieved and independently assessed for eligibility by both reviewers. Any disagreements regarding study eligibility during the screening process were resolved through consensus or by consultation with a third author (A.A.H.). Ultimately, only articles met all inclusion criteria and were selected for final data charting.

### Data charting process

Data extraction was performed independently and in duplicate by two reviewers (G.S.C. and M.B.R.) using a standardized data charting form developed in a spreadsheet program (Microsoft Excel). Prior to implementation, the charting form was piloted on a random sample of three included studies by both reviewers to ensure consistency and clarity in data collection. Any discrepancies identified during data extraction were discussed between the two reviewers until consensus was reached. When consensus could not be reached, the issue was discussed with two additional authors (A.A.H. and E.B.), who assisted in reaching the final decision. The whole data extraction process was supervised by the last two authors.

Authors of the primary studies were not contacted to obtain information that was not reported in the published articles. Variables that could not be identified from the available publications were recorded as ‘Not reported’. Following completion of the data charting, the extracted information was reviewed collectively by all authors to verify its consistency with the predefined review objectives and to guide the synthesis and interpretation of the available evidence.

### Data items

- Study Characteristics: Author, year of publication, and study design (in vitro, ex vivo, or in vivo).
- Restorative Variables: Ceramic material type (e.g., lithium disilicate, zirconia, feldspathic), type of restoration (crown or laminate veneer), and ceramic thickness (measured in millimeters, mm).
- Laser Parameters & Safety Outcomes: Laser model/manufacturer, output power (W), pulse energy (mJ), pulse duration/mode, presence and rate of water/air cooling or irrigation, heat generation during debonding (temperature range in Celsius), debonding time (in seconds), and mechanical outcomes such as shear bond strength (in megapascals, MPa).
- Treatment and control groups: laser and other methods including conventional burs and highspeed motors.

### Synthesis of results

Due to the anticipated heterogeneity of the laser systems, irradiation protocols, restorative characteristics, cooling procedures, and methods used to assess thermal effects, a quantitative synthesis was not considered appropriate. Instead, a narrative synthesis approach was utilized.

Thermal outcomes were considered the primary focus of the synthesis and were summarized according to restoration type (ceramic crowns or laminate veneers), laser system, irradiation parameters, cooling protocol, and restorative characteristics. Temperature changes were reported as described in the original studies, including absolute temperature values and increases relative to baseline when available. Laser parameters, cooling protocols, restoration thickness, and other experimental characteristics were examined to identify factors associated with variations in temperature response.

Additional outcomes, including debonding time, shear bond strength, and failure mode, were summarized when available to provide contextual information regarding debonding performance. The synthesis was used to identify patterns in the reported thermal responses and gaps in the available evidence regarding the thermal safety of erbium laser-assisted ceramic restoration debonding.

## Results

### Selection of sources of evidence

**Fig. 1:**
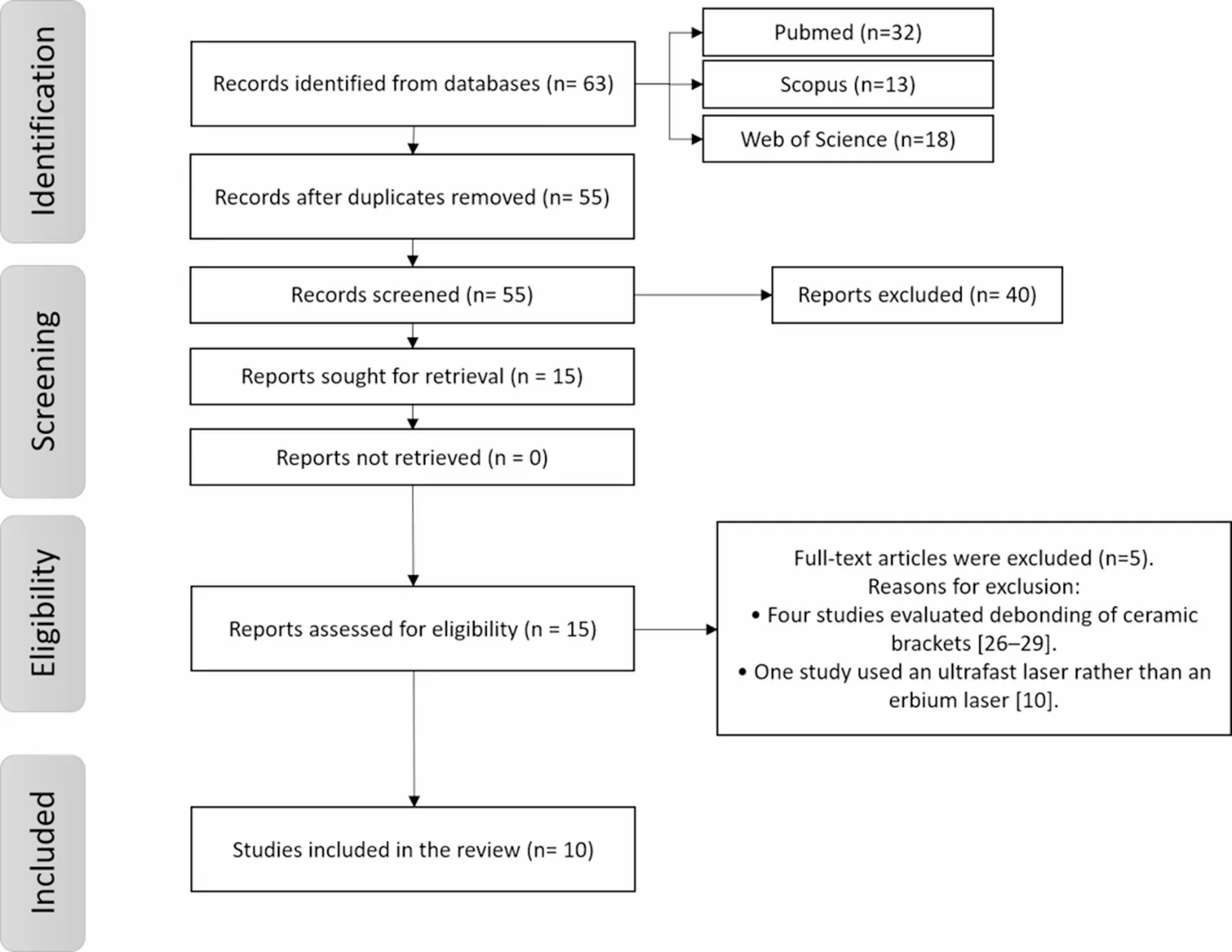
PRISMA-ScR flow diagram of the study selection process

As seen in Fig. 1, in this review study, a total of 63 articles were screened; and 55 remained after removal of duplicate records. Following title and abstract screening, 15 full-text articles were retrieved and assessed for eligibility. However, after a full-text assessment a total of 10 articles were included. Five articles were excluded as they did not meet the predefined inclusion criteria. Specifically, one study did not use an erbium laser for ceramic restoration removal, while the remaining studies did not evaluate erbium laser-assisted debonding/removal of ceramic crowns or laminate veneers with a relevant thermal outcome veneer.

The flow diagram summarizes the study selection process, including records identified through database searching, duplicate removal, title and abstract screening, full-text assessment for eligibility, and final inclusion in the scoping review, following the PRISMA-ScR guidelines.

### Characteristics of sources of evidence

The Er, Cr: YSGG was the most frequently investigated laser, followed by Er: YAG. The studies evaluated the debonding of lithium disilicate and zirconia restorations, including laminate veneers and full-coverage crowns. Considerable heterogeneity was observed regarding ceramic thickness, laser irradiation parameters, and outcome assessment methods. The most reported outcomes included debonding time and intrapulpal temperature change. A detailed summary of the characteristics of the included studies is presented in Tables 2 and 3.

**Table 2:** Characteristics of sources of evidence (highlighting ceramic type and specimens’ thickness)

Author	Year	Study design	Specimen thickness	Ceramic type
Al-Karadaghi & Jawad	2023	In vitro	0.7 ± 0.05 mm	Lithium disilicate (IPS e.max Press HT)
Alikhasi et al.	2019	In vitro	0.7 ± 0.05 mm	Feldspathic/Lithium Disilicate HT / MO
Xiong et al.	2025	In vitro	1.0 mm	PS e.max glass ceramic restoration
Raafat et al.	2025	In vitro	0.5 mm	Lithium disilicate (IPS e.max CAD LT)
Abdulwahhab et al.	2025	In vitro	0.5 mm and 1.0 mm	Leucite-reinforced glass ceramic, IPS Empress
El-Damanhoury et al.	2022	In vitro	0.5 mm and 1.0 mm	Lithium disilicate (IPS e.max CAD)
Gozneli & Sendurur	2023	In vitro	1.0 mm; 1.5 mm; mixed 1–2 mm	Lithium disilicate (IPS e.max CAD)
Deeb et al.	2022	In vitro	Minimum 1 mm ceramic thickness	Prefabricated zirconia crowns (NuSmile^®^)
Rechmann et al.	2015	In vitro	Occlusal: 2.24 ± 0.21 mm; Vestibular: 1.89 ± 0.18 mm; Lingual: 1.81 ± 0.14 mm; Proximal: 1.85 ± 0.14 mm	Lithium disilicate
Al-Karadaghi et al.	2023	In vitro	0.7 ± 0.05 mm	IPS e.max Press HT lithium disilicate

The table summarizes the characteristics and main findings of the studies included in this scoping review

**Table 3:** Characteristics of sources of evidence (highlighting laser parameters and the thermal effect)

Author	Laser	Area	Irrigation	Laser parameter	Time	Thermal effects
Al-Karadaghi & Jawad	Er, Cr: YSGG	8 × 8 mm	Air 40% + Water 20%	3, 3.5, 4, 4.5, and 5 W	60 s	The highest temperature elevation was recorded at 5 W, which increased by 1.7 °C
Alikhasi et al.	Er, Cr: YSGG	4 × 8 mm	Not reported	2.5 W, 25 Hz	Until debond	The intrapulpal temperature increase was less than 1 °C in all groups
Xiong et al.	Er, Cr: YSGG	Not reported	No water mist cooling used	Free running: 3 mJ, 100 Hz, 150 µs and Q-switching running: 3 mJ, 100 Hz, 100 ns	60 s	Nanosecond pulse: 1.8 °C and 2.8 °C Microsecond pulse: 3.4 °C and 5.8 °C
Raafat et al.	Er, Cr: YSGG	4 × 4 × 0.5 mm	1%, 20% or 40% water	15 Hz; 4, 5–6 W	Until debond	The mean intrapulpal temperature increase varied between 1.2–4.00 °C
Abdulwahhab et al.	Er, Cr: YSGG	5 mm	Water 30%; Air 70%	2.5 W; 25 Hz; 60 µs pulse duration	60 s	The maximum temperature increase ranged from 2.6 °C to 2.2 °C
El-Damanhoury et al.	Er: YAG	4 × 6 mm	Water irrigation 40 mL/min; air 40 mL/min	10 Hz; 1.5 W, 3.0–5.4 W; pulse duration 100 µs	Until debond (4 min maximum)	Temperature variation decreased significantly as laser power increased. A low correlation was observed between time and temperature variation
Gozneli & Sendurur	Er: YAG	Full crowns 0.0064 cm^2^	Water/air cooling 2/2 (14 mL/min)	10 Hz; 5.0–5.9 W; pulse duration 100 µs	Until crown debond	The temperature changes in the pulp chamber did not exceed the critical value of 5.5 °C for any sample
Deeb et al.	Er, Cr: YSGG	buccal and lingual surfaces	Water 50% + Air 50%	5.0 Watts, 15 Hz, the fluence in pulse: 22 2 39.32 J/cm, power density: 786.3 W/cm, with 50 water and 50 air, with the Turbo hand-39.32 J/cm, power density: 786.3 W/cm	Until debond (30 s intervals)	Temperature change: rotary handpiece: 0.55 °C and laser: 1.78 °C.
Rechmann et al.	Er: YAG	Not reported	Air-water spray: 7 ml of water per minute	10 Hz, 400 µs at a 590 mJ pulse	85–210 s; average 135 ± 35 s	The temperature rise in the pulp chamber averaged 5.4 °C
Al-Karadaghi et al.	Er, Cr: YSGG	8 × 8 mm	Distilled and deionized water; 60% water, 40% air ratio	3 W; 53 J/cm²; 60 µs (H mode) or 700 µs (S mode)	20, 30, 40, 50–60 s	an exposure time of 50 s and a pulse duration of 60 µs demonstrated superior results for SBS, adhesive remaining index, and temperature values

### Source of funding

This scoping review received no external funding. Among the included studies, two reported financial support: one was supported by the Scientific Research Foundation of the Education Department of Anhui Province of China (grant numbers 2023AH050836 and 2024AH050924) and the Scientific Research Foundation for High-level Talents of Anhui University of Chinese Medicine (grant number DT2300000261); another was partially supported by departmental discretionary funds and partially sponsored by Ivoclar Vivadent AG. The remaining included studies did not report funding sources. The funders of the included studies were not involved in the conduct, analysis, interpretation, or reporting of the present scoping review.

## Discussion

### Intrapulpal temperature

This scoping review aimed to map and synthesize the available evidence regarding the thermal effects of erbium lasers on the dental pulp during the debonding of ceramic crowns and laminate veneers. However, no clinical studies directly evaluated the thermal effects on the dental pulp during ceramic crown or veneer removal were identified. Instead, the available evidence was limited to laboratory studies reporting intrapulpal temperature changes under different irradiation protocols. In previous systematic and scoping reviews, it was found that no clinical studies evaluated the laser effect on ceramic debonding. This finding is consistent with our scoping review, which also identified a lack of clinical studies in the literature searched. Still, the systematic review found that Laser is considered effective, safe and timesaving in ceramic debonding [26, 27].

Overall, the reported intrapulpal temperature increase ranged from approximately 1.2 °C to 5.8 °C, depending on the laser settings and experimental conditions [13, 14, 16–20]. Most studies reported temperature increases below the critical threshold of 5.5 °C traditionally associated with irreversible pulp damage. However, these findings should be interpreted cautiously, as the temperature changes measured in laboratory models do not directly establish the presence or absence of pulpal injury. The biological response to thermal exposure may depend on factors beyond the magnitude of the recorded temperature increase, including the duration of exposure, the characteristics of the experimental model, and the physiological conditions of the pulp [13, 14, 16, 17, 19]. However, one study reported temperature increases exceeding this threshold under inadequate cooling conditions, emphasizing the importance of effective water-cooling during laser irradiation, even though temperature increases below this threshold should not be interpreted as direct evidence of clinical pulpal safety or preservation of pulp vitality [20].

The available evidence consistently demonstrated that intrapulpal temperature is influenced by multiple factors rather than a single variable. Longer irradiation times were associated with greater temperature increases [13, 18], while higher laser power output also contributed to increased heat generation [14, 17]. Some studies reported lower intrapulpal temperature increases with thicker ceramic restorations under the experimental conditions evaluated [14, 16, 19]. Differences in pulse duration and laser operating mode further contributed to the variability observed among studies [10, 13, 18].

Conventional rotary removal provides useful context for interpreting the thermal response associated with erbium laser-assisted debonding veneer. From this perspective, a parallel can be drawn with erbium laser-assisted ceramic veneer removal, in which irradiation time is likewise a critical determinant of intrapulpal temperature increase. However, unlike rotary instrumentation, where heat is generated predominantly by mechanical friction between the diamond bur and dental tissues, laser-assisted debonding involves a more complex interaction among irradiation parameters, the optical properties of the ceramic and resin cement, restoration thickness, and cooling efficiency, indicating that the mechanisms of heat generation and dissipation differ substantially between the two techniques [28].

Only one study directly compared laser-assisted debonding with conventional rotary removal and reported that laser-assisted crown removal produced an intrapulpal temperature increase approximately 2.76 °C higher than rotary instrumentation, with greater temperature increases observed in anterior than posterior teeth [12]. These findings indicate that erbium laser-assisted debonding does not inherently provide a lower thermal response than conventional rotary removal. The observed differences may be related to variations in ceramic restoration thickness, residual dentin thickness, tooth anatomy, or laser and irradiation parameters, although the relative contribution of these factors remains unclear. Therefore, the available evidence does not support a consistent thermal advantage of erbium lasers over conventional rotary removal.

The substantial methodological heterogeneity identified among studies highlights the need for standardized experimental protocols and well-designed clinical investigations to establish evidence-based recommendations for lower intrapulpal temperature increases laser-assisted ceramic restoration debonding [10–20]. Furthermore, all the above highlight that the laser’s thermal advantage is not universal when compared to conventional methods [12]. Nevertheless, it is important to emphasize that pulpal temperature can be controlled by adjusting parameters and scanning strategies, suggesting that thermal response may be influenced by laser calibration, irradiation parameters, cooling conditions, and restorative characteristics; however, their relationship with biological pulpal outcomes remains uncertain [10]. However, differences in reported intrapulpal temperature changes may also reflect methodological variation in temperature measurement rather than differences attributable solely to laser or restorative parameters. Factors such as tooth type and dimensions, residual dentin thickness, baseline temperature, specimen hydration, simulation of pulpal circulation, sensor or thermocouple location, scanning distance, measurement interval, and calibration of the temperature-measuring system may influence heat transfer and the recorded temperature response. The extent to which these variables were controlled or reported varied among the included studies. Therefore, the reported temperature changes should be interpreted within the context of the experimental conditions of each study, and direct comparisons across studies should be made cautiously.

### Ceramic crowns and laminate veneers

In this review, the removal of ceramic crowns and veneers using erbium lasers has proven effective. However, the performance depends on the restorative material type, irradiation parameters, and the use of air/water cooling [11–20]. For laminate veneers, the Er, Cr: YSGG laser successfully removed feldspathic and lithium disilicate ceramics, with mean debonding times of approximately 103–107 s and no significant difference between materials [11]. Conversely, rotary instrumentation was faster than laser-assisted removal for prefabricated zirconia crowns, although laser debonding produced less perceptible damage to the restoration and underlying tooth structure [12]. A more recent study using an ultrafast laser also described rapid, non-invasive veneer removal via carbonization and micro-explosion within the adhesive layer, reducing bond strength to as low as 1.2 MPa, suggesting potential for efficient debonding without affecting the underlying dentin [10]. These findings suggest that, although erbium lasers may not always provide the shortest debonding time, they represent a conservative alternative for restoration removal with the potential to preserve both the restoration and the supporting tooth structure.

The restorative material can influence laser-assisted debonding because ceramic composition and microstructure determine the degree of transmission, absorption, reflection, and scattering of laser energy. For the debonding mechanism to occur, sufficient energy must pass through the ceramic and reach the luting interface, where it can be absorbed by water, hydroxyl groups, residual monomers, and organic components of the cement. Glass ceramics, such as feldspathic porcelain, leucite-reinforced ceramic, and lithium disilicate, generally present greater translucency than polycrystalline zirconia, but their optical behavior also varies according to crystalline content, opacity, shade, and thickness. Therefore, differences among ceramic systems may alter the amount of energy required to degrade the cement and, indirectly, the irradiation time and thermal load imposed on the pulp [12, 13, 18, 20].

Alikhasi et al. [11] compared feldspathic ceramic with high- and medium-opacity lithium disilicate veneer of identical dimensions and reported no significant differences in debonding time, with intrapulpal temperature increases below 1 °C in all groups. Thus, under standardized thickness, cement, laser parameters, scanning method, and cooling conditions, differences in ceramic composition and opacity did not produce measurable differences in thermal response. However, the mechanical behavior differed, since lithium disilicate restorations were more likely to remain intact, whereas the lower fracture resistance of feldspathic ceramic may favor cracking during removal. These findings indicate that ceramic composition may influence restoration´s integrity even when its effect on intrapulpal temperature is limited.

The studies involving lithium disilicate consistently showed that material thickness and restoration design were at least as relevant as the ceramic composition itself. El-Damanhoury et al. [14] found that 1.0-mm veneer required substantially longer irradiation than 0.5-mm veneer when a low power setting was used, and the prolonged exposure was associated with a greater temperature increase. Increasing the output power shortened the removal time and reduced the thermal change, indicating that a low-power, long-duration protocol is not necessarily safer than a higher-power protocol capable of rapidly reaching the debonding threshold. Al-Karadaghi et al. [13] likewise emphasized that ceramic thickness, opacity, resin cement, pulse duration, power, frequency, and exposure time can jointly modify laser transmission and the clinical outcome. Their studies with lithium disilicate veneer showed progressive reductions in bond strength with increasing irradiation time or power, while temperature remained within the adopted safety range.

The results obtained with leucite-reinforced glass ceramic are generally consistent with those observed for other translucent glass ceramics. Abdulwahhab et al. [19] evaluated 0.5- and 1.0-mm leucite-reinforced ceramic discs and reported a slight intrapulpal temperature increase that remained within the conventional safety range after 60 s of Er, Cr: YSGG scanning. Although thinner specimens showed a greater numerical reduction in shear bond strength, the difference between thickness was not statistically significant. These results suggest that leucite-reinforced ceramic permits sufficient laser transmission for weakening the adhesive interface under the tested parameters. Nevertheless, because only one ceramic system, one cement, one power setting, and a fixed exposure time were examined, the results should not be extrapolated to ceramics with different crystalline content or opacity.

Although a previous scoping review found erbium lasers effective in removing all ceramic restorations/appliances from natural teeth and implant abutments [29]. Material-related differences become more evident when glass ceramics are compared with zirconia. Rechmann et al. noted that zirconia may transmit substantially less Er: YAG energy than lithium disilicate because of its higher scattering coefficient, potentially requiring greater incident energy to produce a comparable effect at the cement interface. Nevertheless, Deeb et al. [12] demonstrated that prefabricated zirconia crowns could be removed with an Er, Cr: YSGG laser while preserving crown integrity, although laser removal required considerably longer than rotary removal. The authors attributed this prolonged time not only to zirconia itself, but also to the thicker cement layer required for the passive fit of prefabricated crowns, besides tooth anatomy and retention form. Therefore, the apparent effect of zirconia cannot be separated from differences in restoration design and cement volume [12, 20].

### Laser parameters

Irrigation plays a complex role during laser-assisted ceramic removal because it simultaneously affects heat dissipation and the amount of laser energy reaching the restoration and luting interface. Since water is a major chromophore for erbium lasers, the air–water spray absorbs part of the incident radiation before it reaches the ceramic surface. Consequently, increasing the water percentage may improve cooling and reduce thermal accumulation within the tooth, but it may also attenuate the effective energy delivered to the ceramic–cement interface. Conversely, limited or absent irrigation may increase the energy available for debonding, although this theoretical advantage may be offset by greater heating of the restoration and surrounding dental tissues. This dual behavior helps explain why the effect of irrigation cannot be evaluated independently of output power, exposure time, pulse characteristics, and scanning strategy [11, 17, 18, 20].

The direct comparison between wet and nearly dry Er, Cr: YSGG irradiation performed by Raafat et al. [17] demonstrated that a lower water percentage did not necessarily result in faster or safer debonding. The greatest mean intrapulpal temperature increase was observed with 5 W and 1% water, whereas 6 W combined with 20% water produced both the lowest mean temperature increase and the shortest debonding time. The authors suggested that excessive heating under low-water conditions could alter the ceramic surface through melting and resolidification, thereby reducing its translucency and limiting further laser transmission. Therefore, adequate irrigation may improve debonding efficiency indirectly by preserving the optical pathway through the ceramic, in addition to promoting heat dissipation. These findings indicate that irrigation can influence both the thermal response and the efficiency of the debonding process under the experimental conditions evaluated. However, the results from this specific protocol should not be interpreted as establishing a universally preferred level of irrigation for various laser systems and experimental contexts. Furthermore, smaller temperature increases, in and of themselves, do not demonstrate a reduction in pulpal damage or the preservation of pulpal vitality.

The direction and temperature of the coolant also appear relevant. Rechmann et al. [20] observed that temperature increases exceeding 5.5 °C during Er: YAG-assisted crown removal occurred when the supplementary air–water spray was directed toward the side opposite to that receiving laser irradiation. When the coolant reached the irradiated surface, heat diffusion was more effective, and sustained thermal accumulation was not observed. The authors further proposed that water below room temperature might increase the thermal gradient and enhance heat transfer away from the tooth. Similarly, Gozneli and Sendurur reported that none of the lithium disilicate crowns exceeded the conventional thermal threshold when a constant air–water spray was directed along the same path as the laser irradiation. These results suggest that reporting irrigation merely as “present” or “absent” is insufficient; flow rate, temperature, direction, and spatial relationship to the laser beam should also be considered when comparing thermal outcomes [16, 20].

Nevertheless, cooling requirements may differ according to pulse duration and laser technology. Xiong et al. used water mist with microsecond pulses to limit heat accumulation, whereas nanosecond pulses generated lower intrapulpal temperature changes and were considered capable of reducing the need for external cooling during the initial restoration-debonding stage. Likewise, the ultrafast laser evaluated by Lin et al. produced a restricted heat-affected zone because energy deposition occurred over picosecond-scale pulses, and pulpal temperature could be regulated through adjustments in power, focal position, and scanning strategy. These findings indicate that the influence of irrigation on thermal response may vary according to pulse duration, irradiation time, and laser system, while ultrashort-pulse systems may reduce heat propagation by confining energy deposition more precisely. However, these newer technologies were evaluated under highly controlled laboratory conditions and cannot yet be considered directly interchangeable with conventional Er: YAG or Er, Cr: YSGG clinical protocols [10, 18].

Still, the temperature recorded within the pulp chamber should be interpreted as the final thermal response after laser energy has interacted with the restoration, luting material, and dental tissues. Therefore, this measurement does not directly represent the temperature generated at the ceramic–cement interface or at the external tooth surface. The amount of heat reaching the pulp is influenced by the thermal insulating properties and thickness of the remaining dentin, as well as the duration and magnitude of energy deposition. Greater residual dentin thickness may reduce heat transmission to the pulp, whereas thinner dentin may provide less thermal insulation. Similarly, thermal exposure of the periodontal ligament and surrounding tissues may differ from that measured within the pulp chamber; however, these tissues were not directly evaluated in the included studies. Consequently, the reported intrapulpal temperature changes should be interpreted as an experimental indicator of thermal exposure rather than a direct measure of biological tissue injury.

This review has several limitations. Because all included studies were laboratory-based, the reported intrapulpal temperature changes represent experimental measures of thermal exposure rather than direct evidence of pulpal tissue injury. None of the included studies established whether the recorded temperature increases resulted in histological damage, inflammatory responses, loss of pulp vitality, or subsequent need for endodontic treatment. Consequently, the clinical implications of the reported thermal findings remain uncertain, and the absence of temperature increases exceeding conventionally cited thresholds cannot be taken as evidence that pulpal injury did not occur. Clinical studies incorporating appropriate biological outcomes and follow-up are needed to determine whether the thermal responses observed under laboratory conditions translate into clinically meaningful pulpal effects. Differences in residual dentin thickness, baseline temperature, sensor location, specimen hydration, simulation of pulpal circulation, and other temperature-measurement conditions may have influenced the reported thermal responses. As a scoping review, no formal risk-of-bias or certainty-of-evidence assessment was performed; therefore, this synthesis maps reported experimental findings but does not establish the methodological quality or certainty of evidence regarding pulpal safety. In addition, the search was restricted to selected databases and English-language publications, and gray literature was not systematically searched; therefore, language and publication bias cannot be excluded. These limitations should be considered when interpreting the available evidence and highlight the need for standardized experimental protocols and well-designed clinical studies evaluating both thermal responses and biological pulpal outcomes.

## Conclusions

Current evidence from laboratory and ex vivo studies suggests that the removal of ceramic restorations assisted by erbium lasers can produce temperature increases within the pulp chamber, with the magnitude of the thermal response varying according to laser parameters, cooling protocols, irradiation time, and restorative characteristics. Importantly, the only included study directly comparing laser-assisted and conventional rotary removal reported a greater intrapulpal temperature increase with laser-assisted removal, indicating that erbium lasers do not inherently provide a thermal advantage over conventional instrumentation. However, the absence of clinical studies, together with substantial methodological heterogeneity and limited standardization of experimental protocols and control groups, prevents definitive conclusions regarding pulpal safety or the establishment of evidence-based clinical recommendations. Future well-designed clinical and laboratory studies are needed to optimize laser parameters and support evidence-based recommendations for the safe and predictable removal of ceramic restorations.

## Supplementary Information

Below is the link to the electronic supplementary material.

Supplementary Material 1

## Data Availability

No datasets were generated or analysed during the current study.
